# Deep learning-based time-of-flight (ToF) enhancement of non-ToF PET scans for different radiotracers

**DOI:** 10.1007/s00259-025-07119-z

**Published:** 2025-02-18

**Authors:** Abolfazl Mehranian, Scott D. Wollenweber, Kevin M. Bradley, Patrick A. Fielding, Martin Huellner, Andrei Iagaru, Meghi Dedja, Theodore Colwell, Fotis Kotasidis, Robert Johnsen, Floris P. Jansen, Daniel R. McGowan

**Affiliations:** 1 GE HealthCare, Oxford, UK; 2 GE HealthCare, Waukesha, USA; 3 Wales Research and Diagnostic PET Imaging Centre, Cardiff University, Cardiff, UK; 4 University Hospital of Wales, Cardiff, UK; 5 Department of Nuclear Medicine, Zurich University Hospital, Zurich, Switzerland; 6 Division of Nuclear Medicine, School of Medicine, Stanford University, Stanford, CA USA; 7 Department of Medical Physics and Clinical Engineering, Oxford University Hospitals NHS FT, Oxford, UK; 8 GE HealthCare, Zurich, Switzerland; 9 Department of Oncology, University of Oxford, Oxford, UK

**Keywords:** Deep neural networks, Time of flight, PET, Image quality

## Abstract

**Aim:**

To evaluate a deep learning-based time-of-flight (DLToF) model trained to enhance the image quality of non-ToF PET images for different tracers, reconstructed using BSREM algorithm, towards ToF images.

**Methods:**

A 3D residual U-NET model was trained using 8 different tracers (FDG: 75% and non-FDG: 25%) from 11 sites from US, Europe and Asia. A total of 309 training and 33 validation datasets scanned on GE Discovery MI (DMI) ToF scanners were used for development of DLToF models of three strengths: low (L), medium (M) and high (H). The training and validation pairs consisted of target ToF and input non-ToF BSREM reconstructions using site-preferred regularisation parameters (beta values). The contrast and noise properties of each model were defined by adjusting the beta value of target ToF images. A total of 60 DMI datasets, consisting of a set of 4 tracers (^18^F-FDG, ^18^F-PSMA, ^68^Ga-PSMA, ^68^Ga-DOTATATE) and 15 exams each, were collected for testing and quantitative analysis of the models based on standardized uptake value (SUV) in regions of interest (ROI) placed in lesions, lungs and liver. Each dataset includes 5 image series: ToF and non-ToF BSREM and three DLToF images. The image series (300 in total) were blind scored on a 5-point Likert score by 4 readers based on lesion detectability, diagnostic confidence, and image noise/quality.

**Results:**

In lesion SUV_max_ quantification with respect to ToF BSREM, DLToF-H achieved the best results among the three models by reducing the non-ToF BSREM errors from -39% to -6% for ^18^F-FDG (38 lesions); from -42% to -7% for ^18^F-PSMA (35 lesions); from -34% to -4% for ^68^Ga-PSMA (23 lesions) and from -34% to -12% for ^68^Ga-DOTATATE (32 lesions). Quantification results in liver and lung also showed ToF-like performance of DLToF models. Clinical reader resulted showed that DLToF-H results in an improved lesion detectability on average for all four radiotracers whereas DLToF-L achieved the highest scores for image quality (noise level). The results of DLToF-M however showed that this model results in the best trade-off between lesion detection and noise level and hence achieved the highest score for diagnostic confidence on average for all radiotracers.

**Conclusion:**

This study demonstrated that the DLToF models are suitable for both FDG and non-FDG tracers and could be utilized for digital BGO PET/CT scanners to provide an image quality and lesion detectability comparable and close to ToF.

**Supplementary Information:**

The online version contains supplementary material available at 10.1007/s00259-025-07119-z.

## Introduction

Bismuth germanate (BGO)-based detectors, though not time-of-flight (ToF) capable, were initially preferred in clinical scanners for their photon stopping power (higher sensitivity) and higher photoelectric fraction. However, conventional BGO detectors had limited timing resolution due to their light output and decay time. Recent advancements in silicon photomultipliers (SiPMs) and fast readout electronics have improved BGO’s potential for PET scanners by measuring Cherenkov photons emitted upon 511 keV interaction [1].

Using ToF information, the location of positron-emitting radiopharmaceuticals within the scanner’s field of view (FOV) can be estimated with an uncertainty governed by the scanner’s coincidence timing resolution (CTR). Depending on the activity distribution, this can have a positive impact on contrast recovery at a given noise level [2, 3]. Additionally, the impact of erroneous data correction processes, particularly attenuation correction, is reduced when ToF information is leveraged during reconstruction [4, 5]. Moreover, this localisation leads to higher signal-to-noise ratio (SNR) gain as the contribution of random coincidences will be smaller [6].

The convergence of small low-uptake lesions, that are of paramount importance in early cancer detection, is also influenced by the type of reconstruction algorithm and the selection of its hyperparameters (e.g. number of iterations, regularisation strength, etc.). Ordered subsets expectation maximisation (OSEM) and block sequential regularised expectation maximisation (BSREM [7]) are two model-based iterative reconstruction algorithms widely used with ToF information and point spread function (PSF) modelling for improved diagnostic confidence and lesion detectability [8–10]. PSF modelling is a resolution recovery technique that accounts for the processes that lead to resolution loss in PET [11]. A BSREM algorithm, commercially available in GE HealthCare’s PET/CT scanner as Q.Clear^™^, uses regularisation during reconstruction in order to ensure noise reduction and effective convergence of tracer-avid features. The main limitations of the model-based reconstruction algorithms are (i) they rely on practical assumptions in order to mathematically formulate the characteristics of the PET system, the acquired data, and the image and (ii) the selection of their hyperparameters that depends on a number of factors including the scanner configuration, acquisition protocol and more importantly the patient.

With the recent advancements in artificial intelligence and deep learning (DL), data-driven algorithms have gained significant attention in image reconstruction [12, 13]. These algorithms no longer rely on the assumptions used in model-based algorithm. Instead, they learn a mapping from measured data to image (i.e. direct reconstruction [14–16]), or from one image state to another state (e.g. high-noise to low-noise [17–19] or low-iteration to high-iteration [20]). In Mehranian et al., we trained a deep convolutional neural network (dCNN), named as DLToF, in order to map the images reconstructed by non-ToF BSREM algorithm to their ToF counterparts for improved lesion detectability in ^18^F-FDG oncology PET scans in scanners without ToF capability [21]. Recently, Sanaat et al. [22] also used dCNNs to synthesize ToF sinograms (or images) from non-ToF sinograms (or images) in ^18^F-FDG brain imaging.

In recent years, there has been a tremendous progress in long axial FOV or total-body L[Y]SO-based PET scanners that are now commercially available world-wide. Thanks to their high sensitivity, they allow a reduction in acquisition time or injected activity without impairing image quality, to perform delayed imaging and simultaneous total body dynamic imaging, among others [23, 24]. To provide an affordable long-axial FOV with even higher sensitivity, GE HealthCare (GEHC) has recently introduced a new digital BGO-based PET/CT scanner, Omni Legend^™^, with detector assembly that is scalable up to 128 cm, providing an exceptionally high sensitivity [25].

Given that the benefits of ToF technology manifest in image space and ToF image properties can be emulated by deep learning, the DLToF model [21] has now been deployed in Omni Legend PET systems, commercially branded as Precision DL^™^. This model was trained and deployed for ^18^F-FDG oncology exams only with three different levels of contrast to noise trade off (low: L, medium: M and high: H). In this study, we extended the DLToF models beyond FDG by training them with a range of radiotracers with the hypothesis that with additional tracers the model can be better generalised for four radiotracers of interest: ^18^F-FDG, ^18^F-PSMA, ^68^Ga-PSMA and ^68^Ga-DOTATATE. Hence, these resulting models were considered for oncology, and prostate and neuroendocrine tumours (theranostics) PET imaging.

## Materials and methods

### Data acquisition and processing

**Fig. 1:**
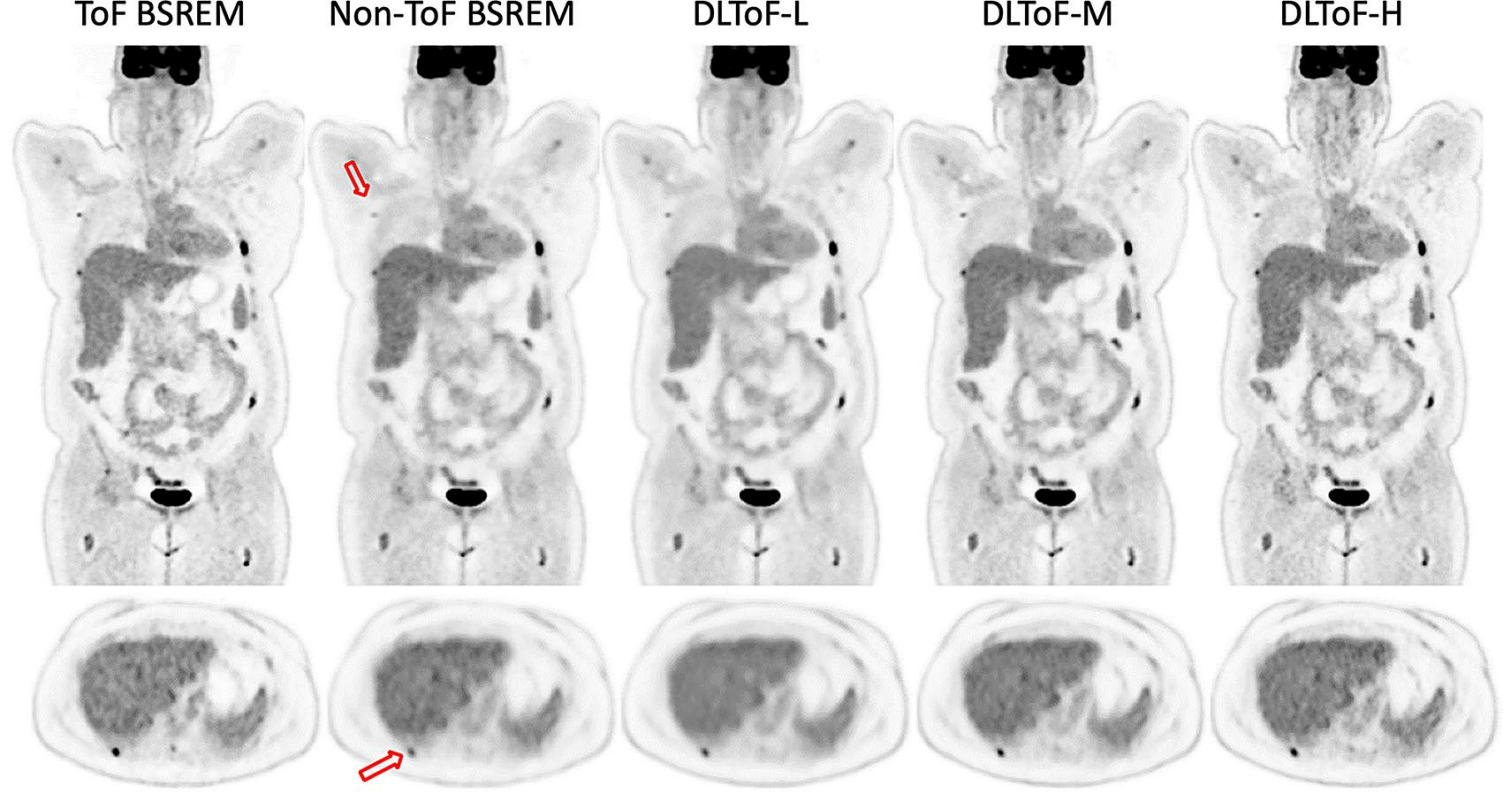
DL-ToF enhancement of an ^18^F-FDG test subject with a BMI of 34.0 kg/m^2^ with an injected activity of 521.3 MBq of scanned on DMI PET/CT scanner. Arrows point to lesions with lower conspicuity in non-ToF BSREM. Display window: 0–5 SUV

The PET list-mode data and CT-based attenuation correction (CTAC) images of a total of 342 exams utilising 8 different tracers scanned on GEHC’s LYSO-based Discovery MI (DMI) ToF PET/CT scanners were retrospectively collected and used for development of multi-tracer DLToF models. Supp Materials Fig. 1shows the distribution of the datasets per tracers. As shown, about 75% of datasets were FDG and the rest were non-FDG. The data were collected from 11 sites in US, Europe and Asia and were split into training (*n* = 309) and validation (*n* = 33) sets.

A total of 60 DMI clinical exams, 15 exams each for a set of 4 primary tracers (^18^F-FDG, ^18^F-PSMA, ^68^Ga-PSMA, ^68^Ga-DOTATATE), were additionally collected to be used as an independent testing set. These exams were used for quantitative evaluation as well as clinical reader studies. Supp. Materials Tables 1 and Supp. Materials Table 2 summarise the distribution of the training, validation and testing sets per site and per tracer. To test the models on a non-ToF PET scanner, four exams were also collected from a GEHC ’s BGO-based Omni Legend scanner for the four primary radiotracers. The testing and validation cases were chosen by two nuclear medicine experts based on the availability of the data as well as pathologically challenging cases with small lesions.

Using training datasets from different clinical sites enhances the generalizability of DLToF models, accommodating different reconstruction parameters and acquisition protocols at each site. The DMI’s PET subsystem offers a nominal ToF resolution of 385 ps, with varying sensitivity based on the number of detector rings (3–6) providing axial FOVs of 15–30 cm. Various scanners and imaging protocols at sites resulted in a range of injected ^18^F-FDG activity (mean ± SD: 315 ± 120 MBq) and scan duration (161 ± 46 s/bed). Patient sizes also varied (body mass index, BMI 27.3 ± 6.0 kg/m²), and uptake times ranged from 82 ± 26 min. Each subject underwent a whole-body CT scan for PET attenuation correction using 100–120 kVp.

Each dataset was reconstructed using the ToF BSREM and non-ToF BSREM algorithm with different regularisation (beta) values depending on the site preferred values. Three models of different strengths, low (L), medium (M) and high (H), were trained in supervised leaning. The training and validation pairs consisted of target ToF and input non-ToF BSREM reconstructions. The strength of each model in terms of image contrast and noise level was defined by adjusting the beta value of target ToF images. Supp. Materials Tables 3 and Supp. Materials Table 4 summarise the beta values chosen for each DL-ToF model, clinical site, and target-input pair for FDG and non-FDG tracers. Each image was reconstructed with a 256 × 256 matrix size and field-of-view 700 mm (x-y pixel size: 2.73 mm, slice thickness: 2.79 mm). Whole-body image volumes used for validation and training were axially divided into equally spaced contiguous 3D patches, each of 50 slices (14 cm).

**Fig. 2:**
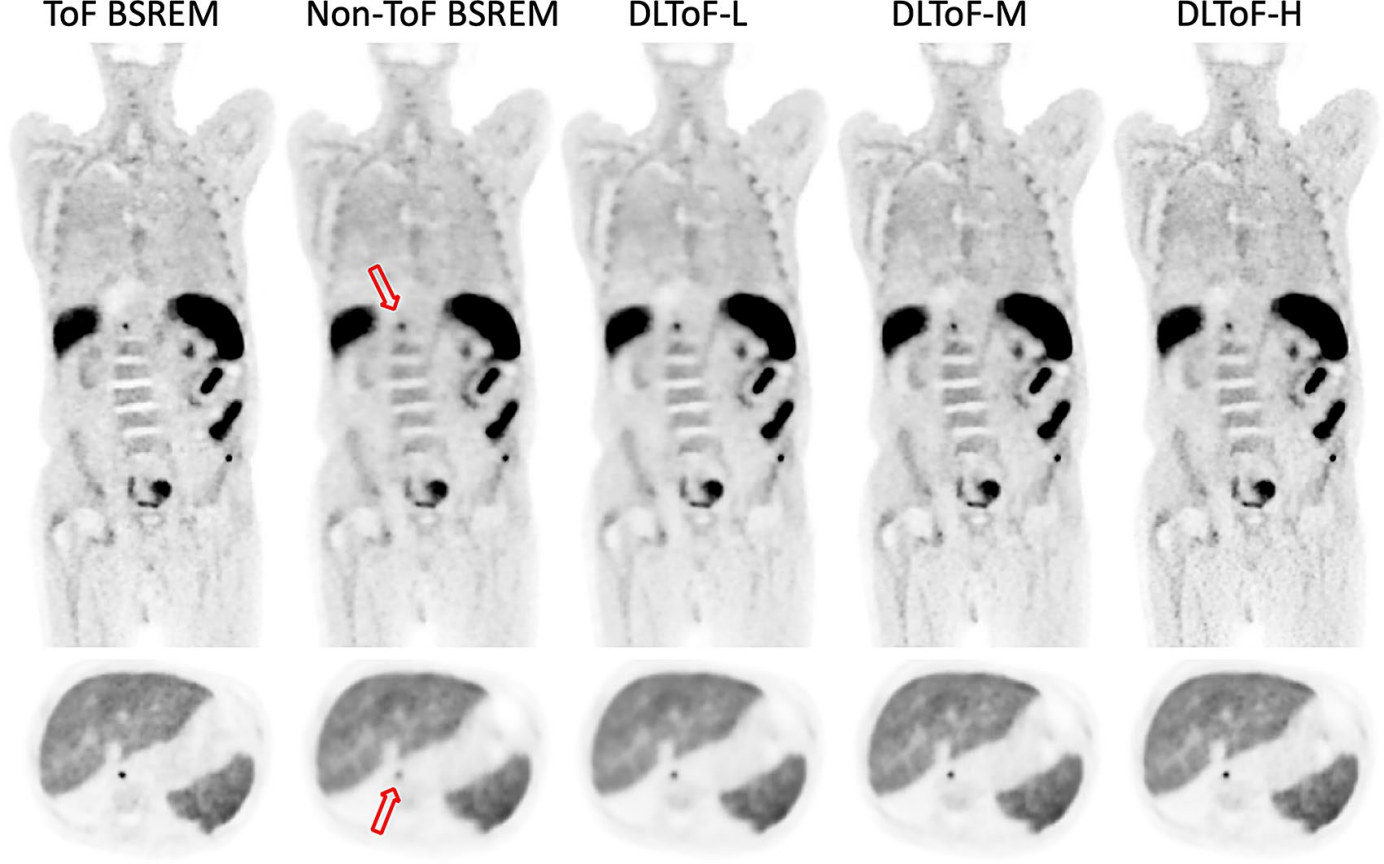
DL-ToF enhancement of a ^18^F-PSMA test subject with a BMI of 23.5 kg/m2 with an injected activity of 346.7 MBq of scanned on DMI PET/CT scanner. Arrows point to lesions with lower conspicuity in non-ToF BSREM. Display window: 0–5 (top) and 0–15 (bottom) SUV

To improve the generalisability of the DLToF models for phantoms, augmented datasets from an anthropomorphic Torso phantom, scanned on a DMI scanner with an axial FOV of 25 cm, were included in the training set. As shown in Supp Materials Fig. 2, the phantom is comprised of liver and lungs with inserted FDG-avid lesions. The phantom list data includes three high-count scans that were augmented to generate extra datasets. Two half-duration and four quarter-duration scan datasets were generated from each one of the three scans. The ToF BSREM and non-ToF BSREM images of each resulting dataset were augmented by a random ± 45° rotation, resulting in a total of 84 phantom datasets. The full, half, and quarter duration datasets were acquired at count levels of 700, 350, and 175 M counts in single-bed-position scans, respectively.

To ensure consistent performance of the models for matrix sizes larger than 256, 50% of all training patches were resampled to larger matrix sizes up to 384 × 384 (voxel size 1.82 × 1.82 × 2.07 mm^3^). Supp. Materials Table 5 summarises the number of training and validation patches using FDG, non-FDG and phantom datasets. The training patches were scaled in standardised uptake value (SUV) and capped at SUV of 20 for both training and inferencing. This value was chosen experimentally and based on the observation that ToF reconstruction primarily impacts small lesions with low SUV [26]. Additionally, this threshold allows a reduction of the dynamic range of input images and as a result minimises artifacts occasionally observed around very hot regions such as bladder or kidneys.

## Model training

**Fig. 3:**
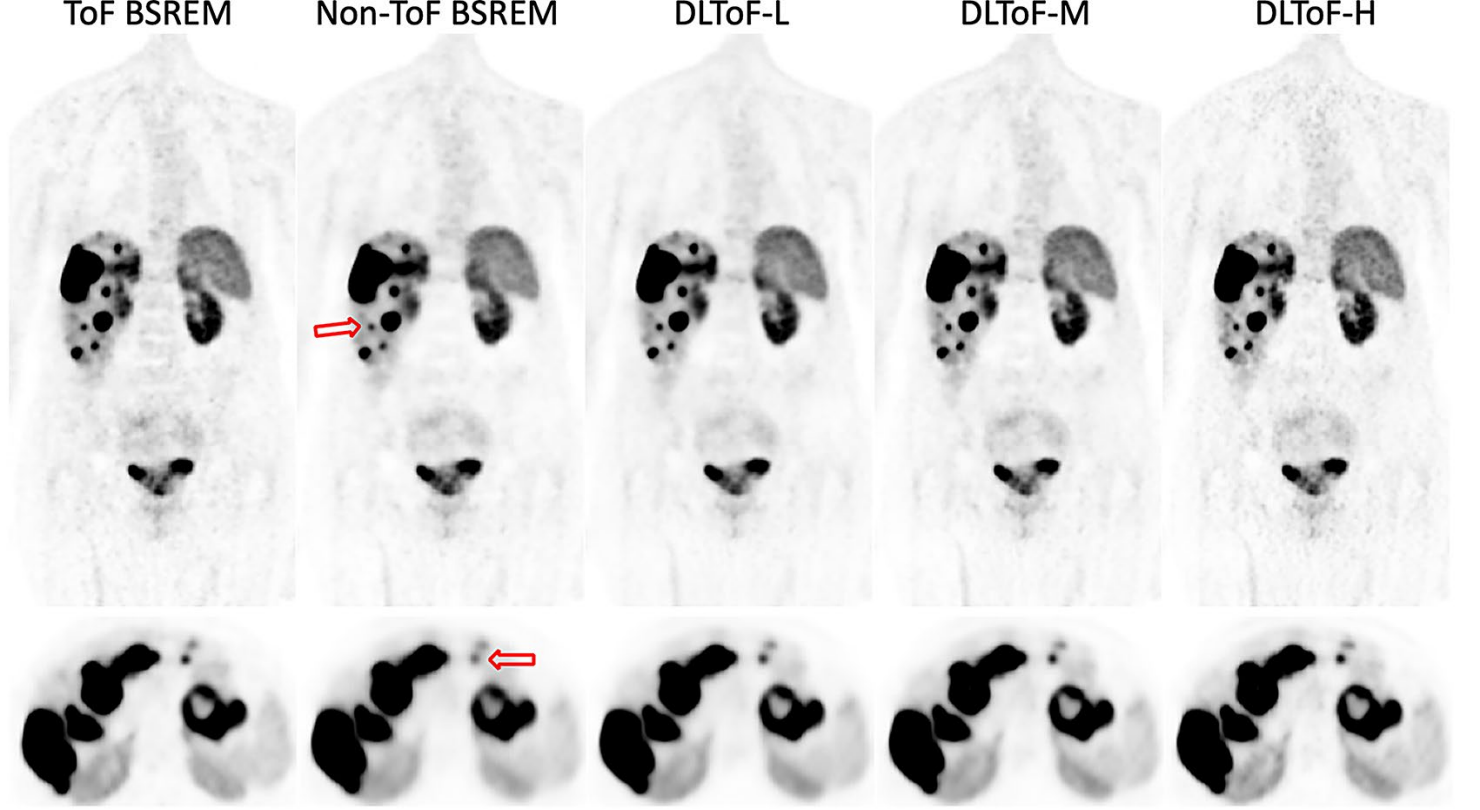
DL-ToF enhancement of an ^68^Ga-DOTATATE test subject with a BMI of 23.1 kg/m^2^ with an injected activity of 187.9 MBq of scanned on DMI PET/CT scanner. Arrows point to lesions with lower conspicuity in non-ToF BSREM. Display window: 0–5 (top) and 0–15 (bottom) SUV

A 3D U-Net network [27] with residual and skip connections was implemented in PyTorch 1.6 (www.pytorch.org) (schematic shown in Supp Materials Fig. 3). DLToF networks were trained in a supervised session where their predicted ToF images were compared to target ToF ones based on mean squared error (MSE) loss function. Supp. Materials Table 6 summarises the network and training hyperparameters that were experimentally optimised. The ADAM algorithm [28] was used to update the networks’ trainable parameters for a maximum of 100 epochs on a workstation with a NVIDIA A40 GPU with 48 GB memory. The validation set was used to monitor the network’s generalisation error to avoid over-fitting. The epoch at which a model had the lowest validation loss and showed no artifacts was chosen as a stopping point.

## Evaluation

The trained DL models were quantitatively evaluated using the testing sets based on SUV measures including lesion SUV_max_ (maximum voxel intensity), SUV_mean_ (mean intensity of voxels) in normal liver and lungs and the noise in the liver using volumes of interest (VOIs) selected per subject. For each subject, 5 VOIs of size 7 × 7 × 7 voxels (~ 7 mL) were defined in the lungs, and 5 similar VOIs in liver. Noise in the liver was calculated as the standard deviation of five VOI mean values. For each subject, up to 5 small lesions were bookmarked and segmented using an adaptive thresholding method (42% of maximum minus minimum SUV in a 7 × 7 × 7 bounding box). The relative difference in SUV values (compared to the target ToF BSREM SUVs), scatter plots and Box-whisker plots were generated. The statistical significance of differences in SUVs was evaluated using unequal variance (Welch) t-test. Additionally, normalised root-mean-square error (NMSE) between SUV of reference ToF images (x) and other images (y) for bookmarked VOIs was calculated by:

$$\:NMSE=\frac{{\sum\:}_{i=1}^{N}{\left({x}_{i}-{y}_{i}\right)}^{2}}{{\sum\:}_{i=1}^{N}{y}_{i}^{2}}$$

where N is the total number of voxels in the VOIs. SUV_mean_ was used for lungs and liver and SUV_max_ for lesions.

Four radiologists, (K.M.B, P.A.F, M.H and A.I), blinded to the image reconstruction method, independently rated all 60 testing sets. Each exam had 5 image series: ToF and non-ToF BSREM, DLToF-L (low), DLToF-M (medium) and DLToF-H (high). The images were evaluated based on three metrics: low-contrast lesion detectability, diagnostic confidence, and image noise/quality based on the Likert scale. The scores were 1 (poor), 2 (satisfactory), 3 (good), 4 (very good), and 5 (excellent) with image noise metrics scored on the same 0–5 scale as described previously [29]. Statistical analysis of the clinical scores was performed with a two-sided paired t-test for each model strength compared to ToF-BSREM (Supp. Materials Table 9) and non-ToF-BRSREM (Supp. Materials Table 10). P-values were Bonferroni corrected.

## Results

### Quantitative analyses

Figures 1, 2 and 3 showcase the performance of the DLToF models in comparison with input non-ToF BSREM and target ToF BSREM images for subjects scanned using ^18^F-FDG, ^18^F-PSMA and ^68^Ga-DOTATATE on a Discovery MI PET/CT scanner. The patients have multiple small lesions that have a lower contrast in the non-ToF image compared to ToF images. The DLToF models improve the conspicuity and contrast of the lesions towards their target ToF images. Since the models were trained to provide different levels of smoothness, the liver noise as well as lesion contrast is different among these models. DLToF-H results in highest contrast enhancement toward ToF while DLToF-L results in the highest noise reduction and DLToF-M provide a balanced contrast enhancement and noise reduction.

Table 1 summarises the quantification results of non-ToF BSREM and DL-ToF methods on the DMI’s 60 testing set (15 exams per 4 radiotracers) for SUV_max_ of the 128 bookmarked lesions, and SUV_mean_ in lungs and liver. The percentage difference from the target ToF BSREM method is provided (mean ± standard deviation). The DLToF method reduces the lesion’s SUV_max_ difference for each radiotracer set depending on their strength or smoothness level. In particular, DLToF-H reduces the average difference from -38.9 to -5.9% for F18-FDG, from -41.8 to -6.7% for F18-PSMA, from -33.5 to -3.7% for ^68^Ga-PSMA and from -33.5 to -12.0% for ^68^Ga-DOTATATE exams. The results in liver and lungs show the differences are relatively small and under 8% for all methods. Supp. Materials Table 7 reports the statistical significance analyses for lesions’ SUV_max_ difference between target ToF BSREM and other methods. The differences between ToF BSREM and particularly DLToF-M and -H are statistically insignificant at the level of 0.05. Supp. Materials Table 8 shows the NMSE performance of the methods in lesions, lungs and liver. As shown, DLToF-H gives rise to the lowest errors for lesions and the error for other regions are relatively low for all methods.

**Fig. 4:**
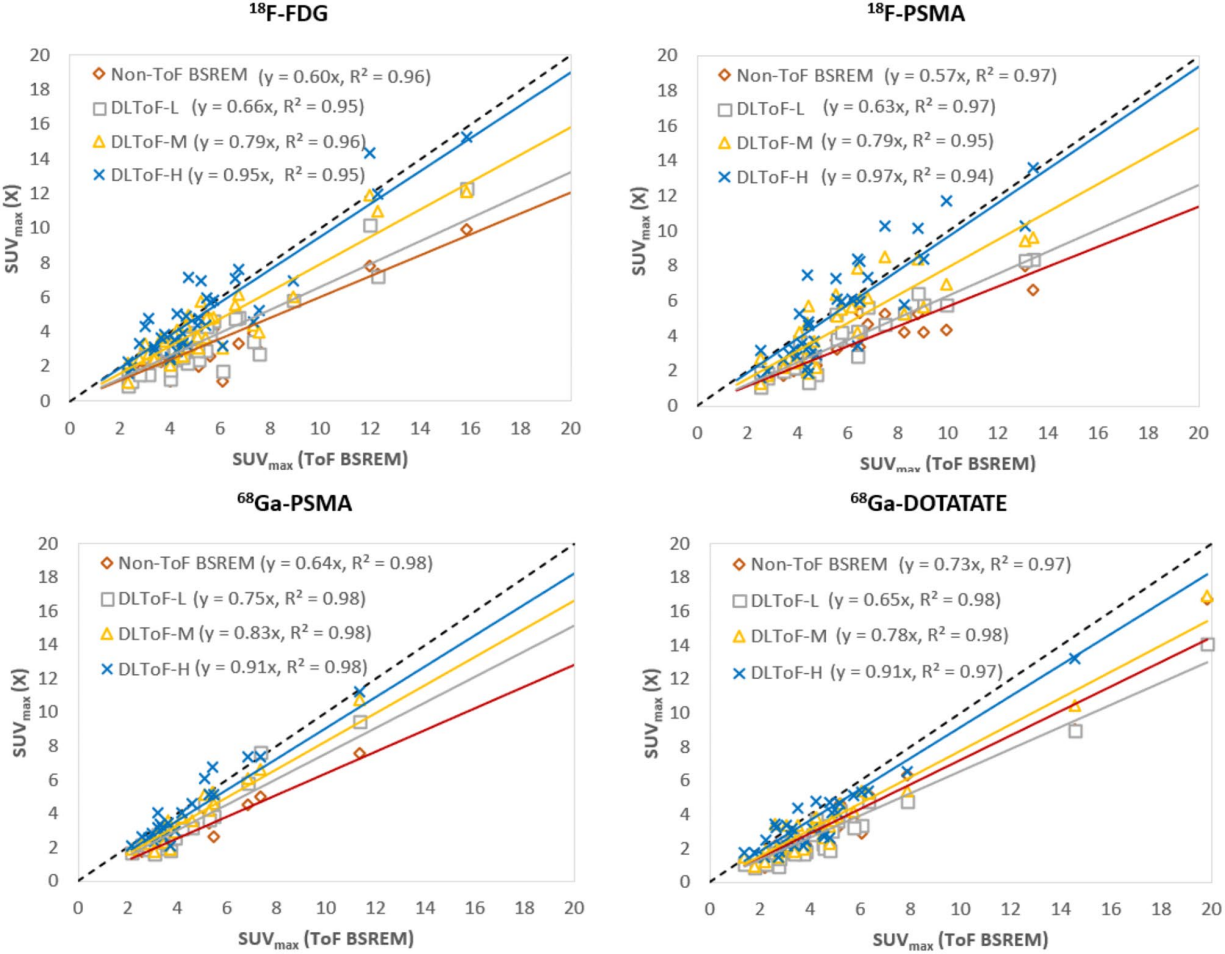
Scatter plots of lesion SUV_max_ or non-ToF BSREM and DL-ToF models compared to ToF BSREM images. Each dot corresponds to a lesion. The dashed line is a line of identity

Figure 4 shows scatter plots of lesion SUV_max_ for non-ToF BSREM and DL-ToF images compared with reference ToF BSREM images. Consistent with Table 1, the non-ToF BSREM method presents a notable deviation of the fitted line (in terms of the slope) in comparison to ToF BSREM method for all radiotracers. As the strength of DLToF is increased, the fitted line slope for DLToF methods gets closer to 1 which demonstrates lesion SUV_max_ enhancement of the input non-ToF images towards ToF. The results show that with DLToF-H the slope of the fitted lines is increased from 0.60 to 0.95 for ^18^F-FDG (+ 58%), from 0.57 to 0.97 for ^18^F-PSMA (+ 70%), from 0.64 to 0.91 (+ 42%) for ^68^Ga-PSMA and from 0.73 to 0.97 (+ 32%) for ^68^Ga-DOTATATE exams.

**Fig. 5:**
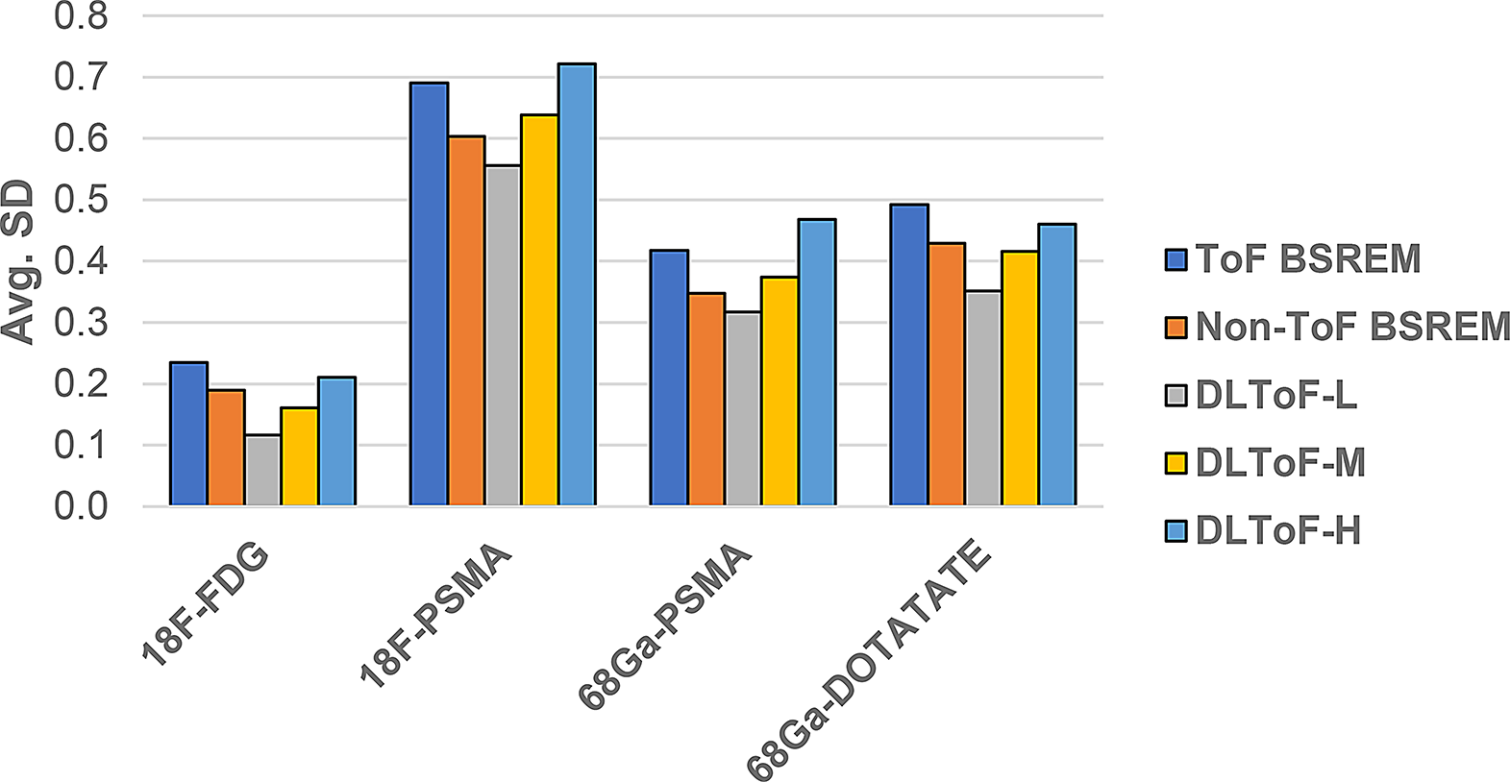
Noise performance of different methods measured as the average of standard deviation (SD) of SUV_mean_ values in liver VOIs in 15 testing exams (5 VOI per exam) per radiotracer

These results demonstrate that as the strength of DLToF is increased from low to high, the lesions’ SUV measure is increased towards their target SUVs. As shown in Fig. 5, the evaluation of liver noise (measured as the average of the standard deviations over 5 liver VOIs per 15 exams for each radiotracer), shows that the DL-ToF models provide different smoothness levels, with DLToF-L resulting in a noise level lower that non-ToF BSREM and DLToF-H resulting in a noise level as high or slightly higher than ToF images. These noise results are consistent with the noise level perceived in Figs. 1, 2 and 3.

**Table 1:** Quantitative performance of the DL-ToF models evaluated on 60 testing exams (15 exams per 4 radiotracers), for lesion SUV_max_, lung SUV_mean_ and liver SUV_mean_ as a percentage difference from ToF BSREM. N is the number of bookmarked lesions. Bold text indicates the least difference in lesion SUV_max_ to ToF BSREM for each radiotracer

Radiotracer	Methods	Lesion SUV_max_(%)	Liver SUV_mean_(%)	Lung SUV_mean_(%)
^18^F-FDG (*n* = 38)	Non-ToF BSREM	-38.9 ± 15.5	4.6 ± 4.3	7.7 ± 13.9
	DLToF-L	-37.0 ± 16.0	2.0 ± 4.3	3.8 ± 13.0
	DLToF-M	-21.9 ± 17.4	3.9 ± 4.2	4.1 ± 13.9
	DLToF-H	**-5.9 ± 24.3**	3.5 ± 3.8	4.1 ± 13.4
^18^F-PSMA (*n* = 35)	Non-ToF BSREM	-41.8 ± 10.0	1.5 ± 4.8	-1.9 ± 12.0
	DLToF-L	-38.0 ± 13.6	0.7 ± 4.4	-2.0 ± 11.0
	DLToF-M	-20.8 ± 22.4	1.4 ± 4.5	-2.9 ± 9.4
	DLToF-H	**-6.7 ± 28.4**	0.4 ± 4.5	-2.1 ± 10.0
^68^Ga-PSMA (*n* = 23)	Non-ToF BSREM	-33.5 ± 10.3	1.9 ± 4.8	2.5 ± 12.2
	DLToF-L	-25.2 ± 12.9	0.8 ± 4.3	-0.7 ± 13.0
	DLToF-M	-15.4 ± 12.0	2.6 ± 4.2	0.8 ± 10.7
	DLToF-H	**-3.7 ± 15.4**	2.2 ± 4.6	2.3 ± 11.6
^68^Ga-DOTATATE (*n* = 32)	Non-ToF BSREM	-33.5 ± 13.0	0.5 ± 4.6	5.4 ± 19.1
	DLToF-L	-37.7 ± 13.2	-0.3 ± 3.7	3.1 ± 14.8
	DLToF-M	-23.9 ± 19.1	0.6 ± 4.0	0.3 ± 15.3
	DLToF-H	**-12.0 ± 22.1**	-0.3 ± 4.1	4.4 ± 15.5

### Clinical reader study

Table 2 shows the average reader scores from four readers for the different PET reconstruction methods (5 image series) for 15 testing exams per 4 tracers (in total 300 images). Supp. Materials Table 9 shows *p*-values for the scores, using pairwise comparisons with respect to ToF BSREM methods. The results show that in terms of low-contrast lesion detectability DLToF-H scores higher than ToF images, on average, for most of the tracers. In terms of image quality and noise, DLToF-L achieves the best scores whereas DLToF-M scores on average higher than ToF images for most of the tracers. These results highlight that one can choose a model that matches preferences as some radiologists prefer sharper but noisier images whereas some prefer smoother ones. Overall, DLToF-M provides a balance between lesion detection and noise reduction and can be a recommended model for most users.

**Table 2:** Clinical image quality scoring from four readers of 15 testing images per radiotracer based on different criteria, mean ± standard deviation. 1 is poor, 5 is excellent. Bold indicates the best (highest) score

Tracer	Method	Low-contrast Lesion Detectability	Diagnostic Confidence	Image Quality
^18^F-FDG	ToF BRSREM	**4.22 ± 0.99**	3.97 ± 1.12	3.33 ± 1.13
	Non-ToF BSREM	3.70 ± 1.08	3.78 ± 1.01	3.98 ± 0.93
	DLToF-L	3.55 ± 1.20	3.67 ± 1.14	**4.82 ± 0.47**
	DLToF-M	4.10 ± 0.75	**4.15 ± 0.73**	4.23 ± 0.65
	DLToF-H	4.12 ± 0.90	3.90 ± 0.99	3.47 ± 0.91
^18^F-PSMA	ToF BRSREM	**4.73 ± 0.58**	**4.40 ± 0.81**	3.70 ± 0.98
	Non-ToF BSREM	3.47 ± 1.05	3.63 ± 1.01	4.45 ± 0.59
	DLToF-L	3.57 ± 1.03	3.72 ± 0.90	**4.83 ± 0.46**
	DLToF-M	4.22 ± 0.80	4.32 ± 0.81	4.40 ± 0.69
	DLToF-H	4.63 ± 0.58	**4.40 ± 0.69**	3.60 ± 0.87
^68^Ga-PSMA	ToF BRSREM	4.43 ± 0.81	4.20 ± 0.82	3.70 ± 0.98
	Non-ToF BSREM	3.55 ± 1.05	3.60 ± 0.98	4.37 ± 0.76
	DLToF-L	3.87 ± 1.07	3.87 ± 1.03	**4.73 ± 0.52**
	DLToF-M	4.25 ± 0.77	**4.23 ± 0.83**	4.28 ± 0.76
	DLToF-H	**4.47 ± 0.70**	4.05 ± 0.79	3.27 ± 0.95
^68^Ga-DOTATATE	ToF BRSREM	4.12 ± 0.92	3.90 ± 0.93	3.43 ± 1.21
	Non-ToF BSREM	3.98 ± 0.89	3.98 ± 0.91	4.02 ± 0.83
	DLToF-L	4.00 ± 1.10	4.03 ± 1.12	**4.75 ± 0.51**
	DLToF-M	4.25 ± 0.86	**4.32 ± 0.85**	4.20 ± 0.73
	DLToF-H	**4.32 ± 0.75**	4.25 ± 0.77	3.57 ± 0.91

## Evaluation on non-ToF PET scanners

**Fig. 6:**
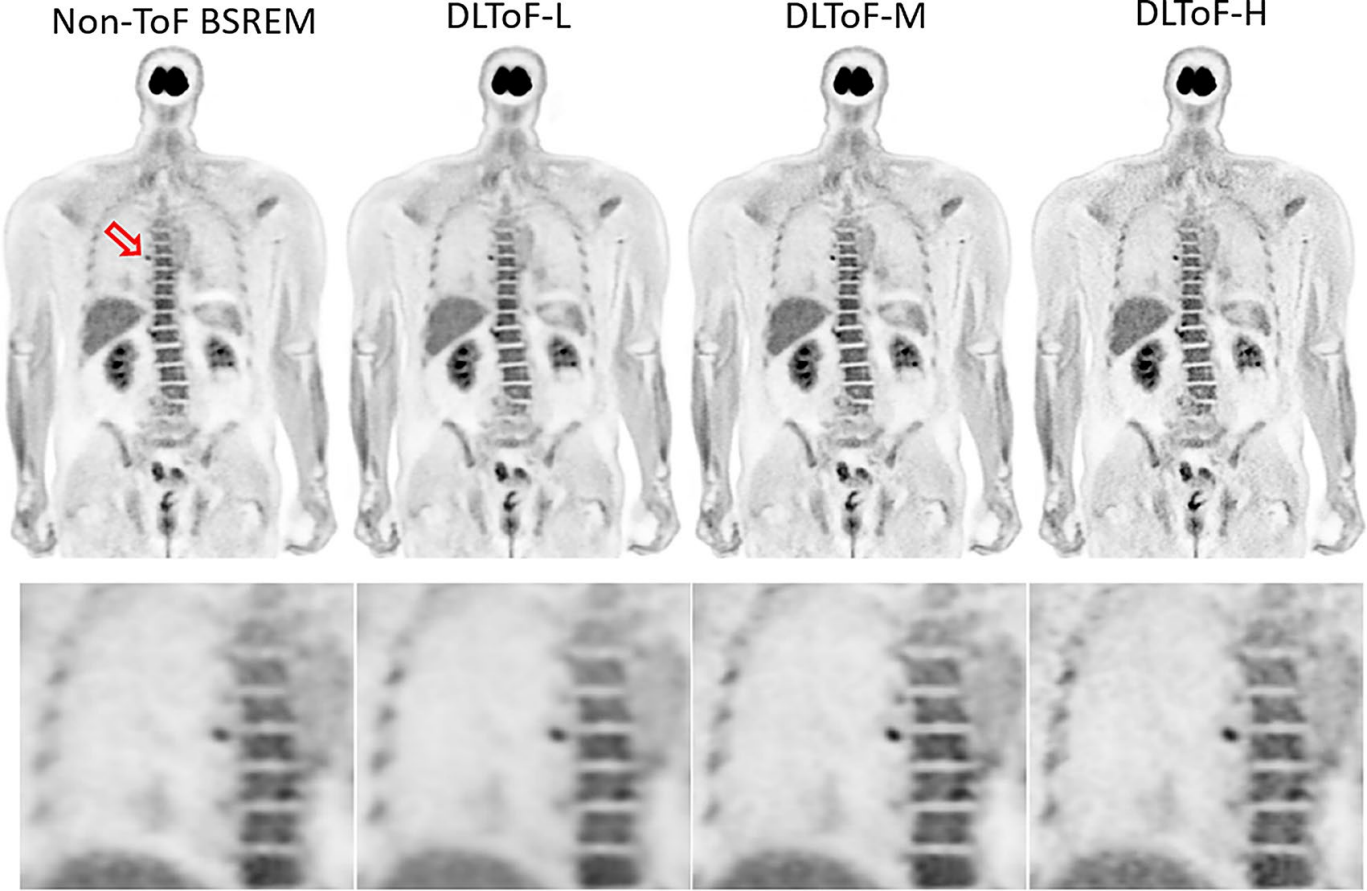
DL-ToF enhancement of a representative ^18^F-FDG test subject with a BMI of 26.3 kg/m^2^ with an injected activity of 346 MBq scanned on a GE Omni Legend PET/CT scanner. Display window: 0–5 SUV

**Fig. 7:**
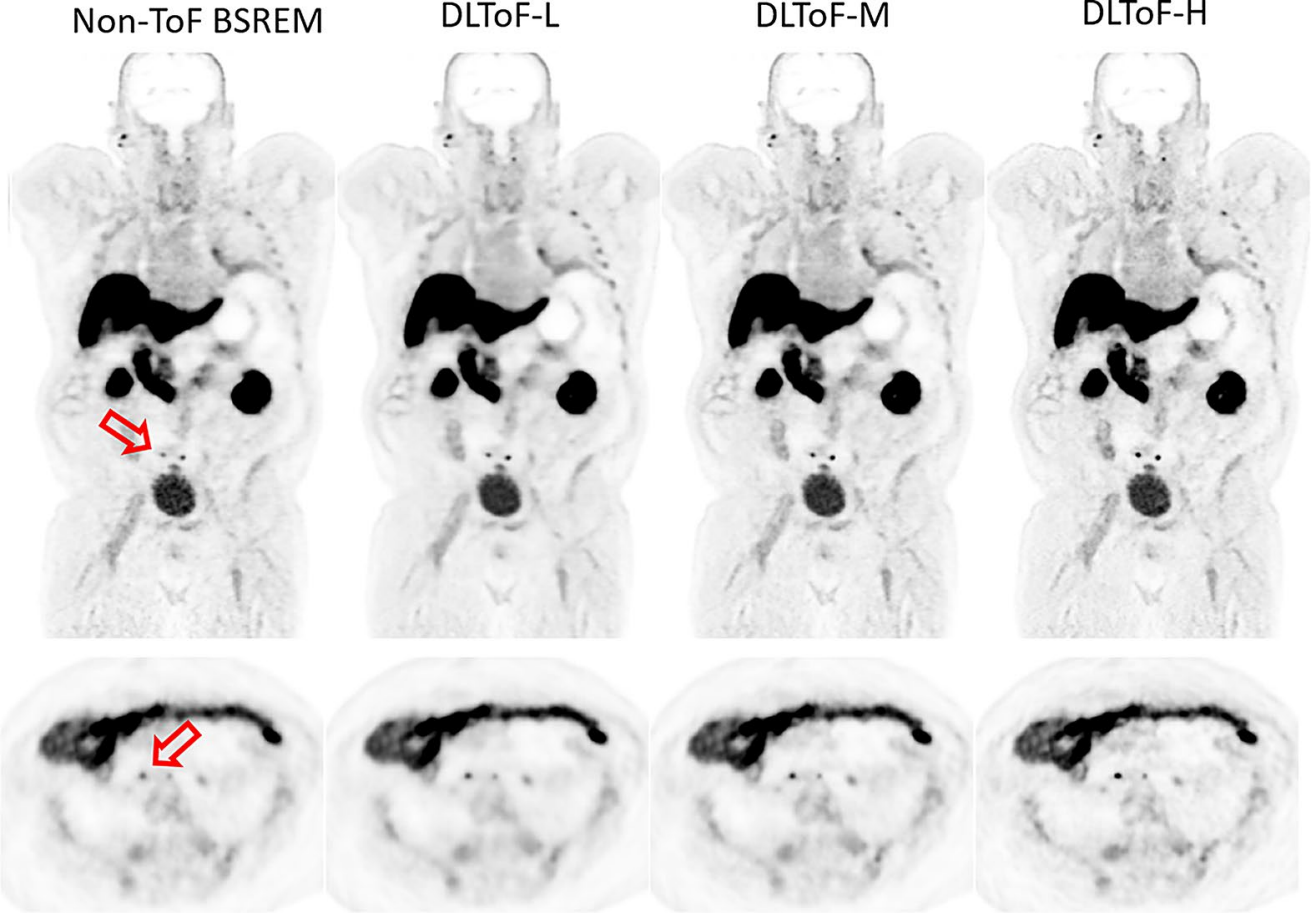
DL-ToF enhancement of a representative ^18^F-PSMA-1007 test subject with a BMI of 39.1 kg/m^2^ with an injected activity of 249 MBq scanned on a GE Omni Legend™ PET/CT scanner. Demonstrating two sub-5 mm PSMA avid retroperitoneal nodes at the L5 level. Display window: 0–6 SUV

To evaluate generalisability of the developed DLToF models on a real non-ToF PET scanner, for each of the studied radiotracers one subject was acquired on an Omni Legend 32 cm PET/CT scanner. In this study, the non-ToF BSREM and DLToF images were evaluated visually. Figures 6 and 7 show examples of ^18^F-FDG and ^18^F-PSMA radiotracers. Supp Materials Fig. 4 and Supp Materials Fig. 5 show two subjects scanned with ^68^Ga-PSMA and ^68^Ga-DOTATATE. As seen, the models improve the conspicuity of small low-contrast lesions especially as the strength of the model is increased towards DLToF-H. Other improvements are higher contrast of small features such as vessel walls and adrenals. These examples show the expected ToF-like enhancement of DLToF models for the image sets from scanners that were not used for training.

## Discussion

In this study, three generalised deep learning models were trained for ToF-like enhancement of features in non-ToF BSREM PET images made with FDG as well as a range of other tracers used for oncology imaging. These models offer three levels of smoothness or model strength to accommodate the wide range of user preferences in terms of contrast and noise levels. This is subsequent to the user chosen regularization parameter of BSREM for a given model to give additional control of the overall image quality. Since the BSREM algorithm, unlike OSEM, is a convergent algorithm while also suppressing noise, this algorithm was used to train DLToF models and shift the learning task to ToF enhancement instead of denoising as much as possible. In this work, the emphasis was on the generalisation of DLToF for a range of radiotracers as well as different image matrix sizes by including different training datasets as well as matrix size data augmentation.

We trained a single model using training datasets from 8 different radiotracers. This design was chosen over multiple models each specific for a tracer or a disease application for two main reasons. Firstly, our initial evaluation (presented in Supp Materials Figs. 6–9) showed that our previous FDG-only DLToF models are generalised to an extent that can provide ToF-like enhancements for non-FDG exams. Therefore, adding non-FDG data to the FDG training pool (75% FDG and 25% non-FDG) is aligned with fine-tuning or transfer learning schemes. Secondly, the lower abundance of non-FDG exams in nuclear medicine departments limits the number of non-FDG datasets for training of tracer specific models. Similarly, Sanaat et al. [30] trained a single multi-tracer DL model for partial volume correction in brain PET imaging using four different radiotracers. Supp Materials Table 11 compares the quantitative performance of the DL-ToF multi-tracer with the FDG single-tracer version for our 15 FDG test exams. As shown for the cohort of 38 lesions used in this comparison, the multi-tracer improves upon the single-tracer version which can be attributed to the larger training sets of the new model. Additionally, six FDG exams were selected for blinded reading by one of our readers (KMB) comparing the two versions in terms of diagnostic confidence, lesion detection and overall image quality. The results presented in Supp Materials Fig. 10 show a comparable performance between these models for this subset of exams.

Our quantitative evaluation in Table 1 indicates that the non-ToF BSREM algorithm results in about -37% error in SUV_max_ for target lesions averaged across all four radiotracers with respect to the reference ToF BSREM algorithm whereas DLToF-H reduces the error to about -7%. This improved quantitative performance is consistent with the clinical reader study in Table 2 which demonstrated that DLToF-H notably increased lesion detection compared to ToF BSREM. For the ^18^F-FDG, ^68^Ga-PSMA and ^68^Ga-DOTATATE testing sets, DLToF-H scored slightly higher than ToF BSREM (~ 2% on average) while for ^18^F-PSMA testing set ToF BSREM score slightly higher (~ 2%) for lesion detectability. These results showed that DLToF with the highest strength performs as well as ToF BSREM for lesion detectability. Noise measurements in Fig. 5 show that this model results in an average noise level across all radiotracers as high as ToF BSREM (0.47 for DLToF-H versus 0.46 for ToF BSREM). Compared to the quantitative performance of DLToF H and L models, and their noise performance, DLToF-M provides a balanced performance which can explain the on-average higher diagnostic confidence score of this model. Our additional lesion SUV_max_ quantitative analyses in terms of scatter plots in Fig. 4 and NRMSE in Supp. Materials Table 8 also showed that DLToF-H presents the best match to ToF BSREM and has the lowest NRMSE for all tracers. Our qualitative evaluation of the DLToF models, on Omni PET/CT scanner testing sets, showed that the models improve image quality and lesion conspicuity as expected for a non-ToF BSREM image. Quantitative evaluations using a large Omni dataset in terms of feature SUV change from the baseline non-ToF BSREM is out of the scope of this study and will be performed in future work.

While this study has some limitations, they do not significantly affect the validity of the conclusions. Our testing sets do not include randomly selected exams (i.e. combination of normal/abnormal) but rather patients were chosen with small and low-contrast lesions or those that were completely missed in non-ToF BSREM images. Therefore, our results might be biased to emphasize the gap between ToF and non-ToF reconstructions. Despite this limitation, the clinical reader study which was targeted to challenging cases, demonstrated successful application of the deep learning models which were generally the most preferred in terms of diagnostic confidence, reflecting a combination of lesion detectability and image quality. Another limitation is that the readers were shown at the same time all 5 image series (blinded and in random order) of a subject at a time. This might bias the scoring of the images, although it was considered advantageous, to facilitate the detection of false positive or missing lesions by comparing images concurrently. Another limitation that leaves room for future work, is that a data sufficiency experiment was not performed for the selection of the proportion of multi-tracer datasets. Future work includes the quantitative and clinical evaluation of the trained models for the same set of oncology and other radiotracers as used in this study but for Omni Legend™ PET/CT scanners. Since with BGO based scanners there’s no ToF image as a reference, the quantitative evaluations should include ground truth images using inserted lesions. Another future direction is to evaluate the models presented in this work for dynamic whole-body imaging using DMI and Omni datasets.

### Conclusion

This study extended our previously ^18^F-FDG-only DLToF models to a range of tracers beyond FDG including those primarily used in theranostics PET imaging (i.e. PSMA and DOTATATE). The results of our quantitative analyses as well as clinical reader study demonstrated that the proposed multi-tracer models (deployed in three strength levels) improve the feature quantification, image sharpness and diagnostic value. Depending on the model strength, DLToF-H showed highest improvement in lesion detection and quantification, DLToF-L resulted in the greatest noise reduction and DLToF-M presented a balanced performance for lesion detection and noise reduction, hence achieved the best diagnostic confidence score across all radiotracers.

## Electronic supplementary material

Below is the link to the electronic supplementary material.

Supplementary Material 1

## Data Availability

Data is available by reasonable request to the corresponding author.

## Code availability

The code used in the preparation of this manuscript is not available for public distribution.
