# Supplementary material for: Deep learning-based time-of-flight (ToF) enhancement of non-ToF PET scans for different radiotracers
Source: Eur J Nucl Med Mol Imaging. 2025 Feb 18;52(8):2968–78. doi: 10.1007/s00259-025-07119-z (PMC12162374; doi:10.1007/s00259-025-07119-z)
Supplement: Supplementary file 1 — Supplementary Material 1 [file 259_2025_7119_MOESM1_ESM.docx]

**Supplementary Tables**

Supp. Materials **Table 1** Distribution of all DMI training, validation and testing set per site.

| **Site (Location)** | **Training** | **Validation** | **Testing** |
| --- | --- | --- | --- |
| 1 (Asia) | 8 | 0 | 0 |
| 2 (Asia) | 6 | 2 | 7 |
| 3 (USA) | 22 | 3 | 2 |
| 4 (USA) | 0 | 0 | 1 |
| 5 (USA) | 29 | 7 | 5 |
| 6 (USA) | 87 | 11 | 11 |
| 7 (USA) | 87 | 2 | 7 |
| 8 (USA) | 1 | 0 | 0 |
| 10 (Europe) | 0 | 1 | 0 |
| 11 (Europe) | 69 | 7 | 27 |
| **Total** | **309** | **33** | **60** |

Supp. Materials **Table 2** Distribution of all DMI training, validation and testing set per tracer.

| **Radiotracer** | **Training** | **Validation** | **Testing** |
| --- | --- | --- | --- |
| ^18^F FDG | 232 | 19 | 15 |
| ^18^F PSMA | 15 | 4 | 15 |
| ^68^Ga PSMA | 18 | 4 | 15 |
| ^68^Ga DOTATATE | 30 | 3 | 15 |
| ^68^Ga RM2 | 7 | 2 | 0 |
| ^18^F Fluciclovine | 5 | 1 | 0 |
| ^18^F FDOPA | 1 | 0 | 0 |
| ^18^F FES | 1 | 0 | 0 |
| **Total** | **309** | **33** | **60** |

Supp. Materials **Table 3** FDG training datasets. The beta values chosen for the target QCFX images of three levels of PDL (H: high, M: Medium, L: Low) together with the beta values of the input QCHD images. All the datasets are full duration.

| **Source site** | **Target beta (QCFX)** | | | **Input beta (QCHD)** | | |
| --- | --- | --- | --- | --- | --- | --- |
|  | H | M | L | H | M | L |
| 1 | 250 | 550 | 1050 | 200, 350, 500 | 200, 350, 500, 750 | 350, 500, 750 |
| 2 | 150 | 350 | 850 | 200, 350, 500 | 200, 350, 500, 750 | 350, 500, 750 |
| 3 | 250 | 500 | 1000 | 200, 350, 500 | 200, 350, 500, 750 | 350, 500, 750 |
| 4 | 250 | 550 | 1050 | 200, 350, 500, 550 | 200, 350, 500, 550, 750 | 350, 500, 550, 750 |
| 5 | 500 | 700 | 1000 | 500, 700, 1000 | 500, 700, 1000 | 500, 700, 1000 |
| 6 | 325 | 450 | 700 | 325, 450, 700 | 325, 450, 700 | 325, 450, 700 |
| Average | 287.5 | 516.7 | 941.7 |  |  |  |

Supp. Materials **Table 4** Non-FDG training datasets. The beta values chosen for the target QCFX images of three levels of PDL (H: high, M: Medium, L: Low) together with the beta values of the input QCHD images. All the datasets are full duration. The values in parentheses reflect that beta values varied per tracers.

| **Source site** | **Target beta (QCFX)** | | | **Input beta (QCHD)** |
| --- | --- | --- | --- | --- |
|  | H | M | L | All models |
| 2 | 250 | 350 | 500 | 550, 425, 350, 300,250 |
| 3 | (350)  (350)  (700) | (800)  (650)  (1000) | (1000)  (800)  (1300) | (1000, 850, 650, 450, 350)  (1000, 850, 650, 450, 350)  (1300, 1150, 1000, 850, 700) |
| 4 | (700)  (250) | (1000)  (450) | (1300)  (850) | (1300, 1150, 1000, 850, 700)  (850, 600, 450, 325, 250) |
| 6 | (350)  (250) | (700)  (450) | (1000)  (600) | (1000, 850, 700, 500, 350)  (850, 600, 450, 325, 250) |
| 7 | 250 | 400 | 550 | 700, 550, 400, 325, 250 |
| 8 | (350)  (250) | (700)  (500) | (1000)  (700) | (1000, 850, 700, 500, 350)  (850, 700, 500, 350, 250) |

Supp. Materials **Table 5**. Number of patch pairs per dataset including data augmentation.

| **Dataset** | **Clinical Data**  **(FDG)** | **Clinical Data**  **(non-FDG)** | **Phantom Data** | **Total** |
| --- | --- | --- | --- | --- |
| Training Set | 9834 | 4929 | 773 | 15536 |
| Validation Set | 690 | 599 | 0 | 1331 |

Supp. Materials **Table 6**. DLToF Model Hyperparameters

| **Hyper Parameter** | **Value** |
| --- | --- |
| Number of resolution levels | 4 |
| Skip connections | 4 |
| Number of kernels at 1^st^ layer | 64 |
| Kernel size | 3×3×3 |
| Batch size | 8 |
| Number of epochs | 100 |
| Dropout factor | 0.2 |
| SUV threshold | 20 |
| Learning rate | 0.001 |
| Optimisation algorithm | Adam |
| Loss function | MSE |
| # training parameters | 42,944,832 |

Supp. Materials **Table 7**. Statistical significance analysis between lesion SUV_max_ of target ToF BSREM and different methods using a two-tail t-test with two sample of unequal variances (Welch t-test). If p-value > 0.05 it means the two group’s means are not significantly different.

| **Radiotracer** | **Methods** | ***p*-value** |
| --- | --- | --- |
| ^18^F-FDG (n=38) | Non-ToF BSREM | 0.000 |
|  | DLToF-L | 0.002 |
|  | DLToF-M | 0.063 |
|  | DLToF-H | 0.640 |
| ^18^F-PSMA (n=35) | Non-ToF BSREM | 0.000 |
|  | DLToF-L | 0.000 |
|  | DLToF-M | 0.061 |
|  | DLToF-H | 0.725 |
| ^68^Ga-PSMA (n=23) | Non-ToF BSREM | 0.083 |
|  | DLToF-L | 0.252 |
|  | DLToF-M | 0.472 |
|  | DLToF-H | 0.828 |
| ^68^Ga-DOTATATE (n=32) | Non-ToF BSREM | 0.082 |
|  | DLToF-L | 0.034 |
|  | DLToF-M | 0.186 |
|  | DLToF-H | 0.539 |

Supp. Materials **Table 8**. Quantitative performance of the DL-ToF models evaluated on 60 testing exams (15 exams per 4 radiotracers), for lesion SUV_max_, lung SUV_mean_ and liver SUV_mean_ as NRMSE(%) from ToF BSREM.

| **Radiotracer** | **Methods** | **Lesion SUV_max_ (%)** | **Liver SUV_mean_ (%)** | **Lung SUV_mean_ (%)** |
| --- | --- | --- | --- | --- |
| ^18^F-FDG (n=38) | Non-ToF BSREM | 17.5 | 0.4 | 3.0 |
|  | DLToF-L | 13.8 | 0.2 | 2.1 |
|  | DLToF-M | 6.8 | 0.3 | 2.5 |
|  | DLToF-H | **4.5** | 0.3 | 2.1 |
| ^18^F-PSMA (n=35) | Non-ToF BSREM | 19.3 | 0.2 | 1.4 |
|  | DLToF-L | 14.6 | 0.2 | 1.1 |
|  | DLToF-M | 7.9 | 0.2 | 0.9 |
|  | DLToF-H | **6.0** | 0.2 | 1.0 |
| ^68^Ga-PSMA (n=23) | Non-ToF BSREM | 13.3 | 0.0 | 1.0 |
|  | DLToF-L | 7.1 | 0.1 | 1.4 |
|  | DLToF-M | 3.7 | 0.0 | 1.0 |
|  | DLToF-H | **2.6** | 0.0 | 0.9 |
| ^68^Ga-DOTATATE (n=32) | Non-ToF BSREM | 9.1 | 0.1 | 2.4 |
|  | DLToF-L | 12.8 | 0.1 | 1.7 |
|  | DLToF-M | 6.3 | 0.1 | 1.8 |
|  | DLToF-H | **2.8** | 0.1 | 2.1 |

Supp. Materials **Table 9**. Bonferroni corrected p-value results for the clinical reader scores compared to ToF BSREM scores.

| **Tracer** | **Method** | **Low-contrast Lesion Detectability** | **Diagnostic Confidence** | **Image  Quality** |
| --- | --- | --- | --- | --- |
| ^18^F-FDG | Non-ToF BSREM | 0.097 | 1.000 | <0.001 |
|  | DLToF-L | 0.003 | 1.000 | <0.001 |
|  | DLToF-M | 1.000 | 1.000 | <0.001 |
|  | DLToF-H | 1.000 | 1.000 | 1.000 |
| ^18^F-PSMA | Non-ToF BSREM | <0.001 | <0.001 | <0.001 |
|  | DLToF-L | <0.001 | <0.001 | <0.001 |
|  | DLToF-M | 0.004 | 1.000 | <0.001 |
|  | DLToF-H | 1.000 | 1.000 | 1.000 |
| ^68^Ga-PSMA | Non-ToF BSREM | <0.001 | <0.001 | <0.001 |
|  | DLToF-L | 0.008 | 0.320 | <0.001 |
|  | DLToF-M | 1.000 | 1.000 | <0.001 |
|  | DLToF-H | 1.000 | 1.000 | <0.001 |
| ^68^Ga-DOTATATE | Non-ToF BSREM | 1.000 | 1.000 | <0.001 |
|  | DLToF-L | 1.000 | 1.000 | <0.001 |
|  | DLToF-M | 1.000 | 0.166 | <0.001 |
|  | DLToF-H | 1.000 | 0.214 | 1.000 |

Supp. Materials **Table 10** Bonferroni corrected p-value results for the clinical reader scores compared to non-ToF BSREM scores.

| **Tracer** | **Method** | **Low-contrast Lesion Detectability** | **Diagnostic Confidence** | **Image  Quality** |
| --- | --- | --- | --- | --- |
| ^18^F-FDG | DLToF-L | 1.000 | 1.000 | <0.001 |
|  | DLToF-M | 0.018 | 0.048 | 0.842 |
|  | DLToF-H | 0.477 | 1.000 | <0.001 |
| ^18^F-PSMA | DLToF-L | 1.000 | 1.000 | 0.030 |
|  | DLToF-M | <0.001 | <0.001 | 1.000 |
|  | DLToF-H | <0.001 | <0.001 | <0.001 |
| ^68^Ga-PSMA | DLToF-L | 0.050 | 0.554 | 0.016 |
|  | DLToF-M | <0.001 | <0.001 | 1.000 |
|  | DLToF-H | <0.001 | 0.015 | <0.001 |
| ^68^Ga-DOTATATE | DLToF-L | 1.000 | 1.000 | <0.001 |
|  | DLToF-M | 1.000 | 0.387 | 1.000 |
|  | DLToF-H | 0.728 | 1.000 | 0.004 |

Supp. Materials **Table 11** Quantitative performance of the DL-ToF multi-tracer (MT) and DL-ToF FDG-only single-tracer (ST) models evaluated on 15 ^18^F-FDG testing exams, for lesion SUV_max_, lung SUV_mean_ and liver SUV_mean_ as a percentage difference from ToF BSREM.

| **Methods** | **Lesion SUV_max_(%)** | **Liver SUV_mean_(%)** | **Lung SUV_mean_(%)** |
| --- | --- | --- | --- |
| Non-ToF BSREM | -38.9 ± 15.5 | 4.6 ± 4.3 | 7.7 ± 13.9 |
| DLToF-L (MT) | -37.0 ± 16.0 | 2.0 ± 4.3 | 3.8 ± 13.0 |
| DLToF-M (MT) | -21.9 ± 17.4 | 3.9 ± 4.2 | 4.1 ± 13.9 |
| DLToF-H (MT) | -5.9 ± 24.3 | 3.5 ± 3.8 | 4.1 ± 13.4 |
| DLToF-L (ST) | -41.2 ± 14.5 | 3.3 ± 12.1 | 2.9 ± 4.1 |
| DLToF-M (ST) | -26.8 ± 16.3 | 3.8 ± 4.1 | 3.3 ± 12.0 |
| DLToF-H (ST) | -10.3 ± 20.3 | 4.7 ± 11.7 | 3.0 ± 3.9 |

**Supplementary Figures**

Supp Materials **Figure** 1 The proportion of different radiotracers in the clinical exams used for training of the DLToF models.


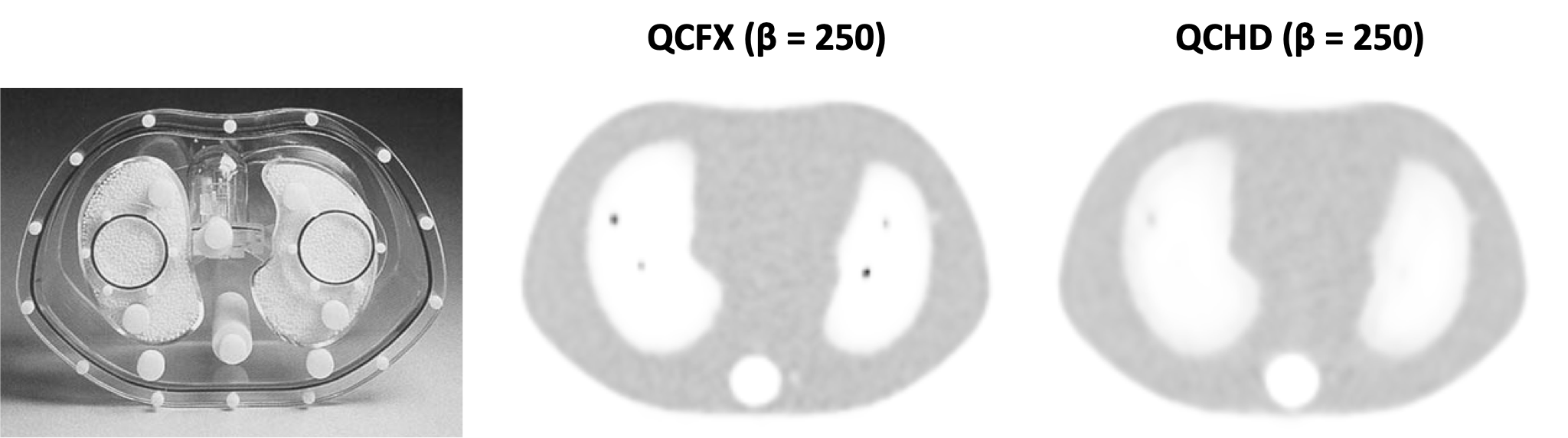


Supp Materials **Figure** 2 The anthropomorphic torso phantom photograph and its PET images reconstructed with (QCFX) and without (QCHD) ToF BSREM algorithm in a DMI PET/CT scanner. The beta is the regularisation parameter used for the reconstructions.

Supp Materials **Figure 3** The architecture of the 3D UNET convolutional network, with residual and skip connections, used in DLToF models.


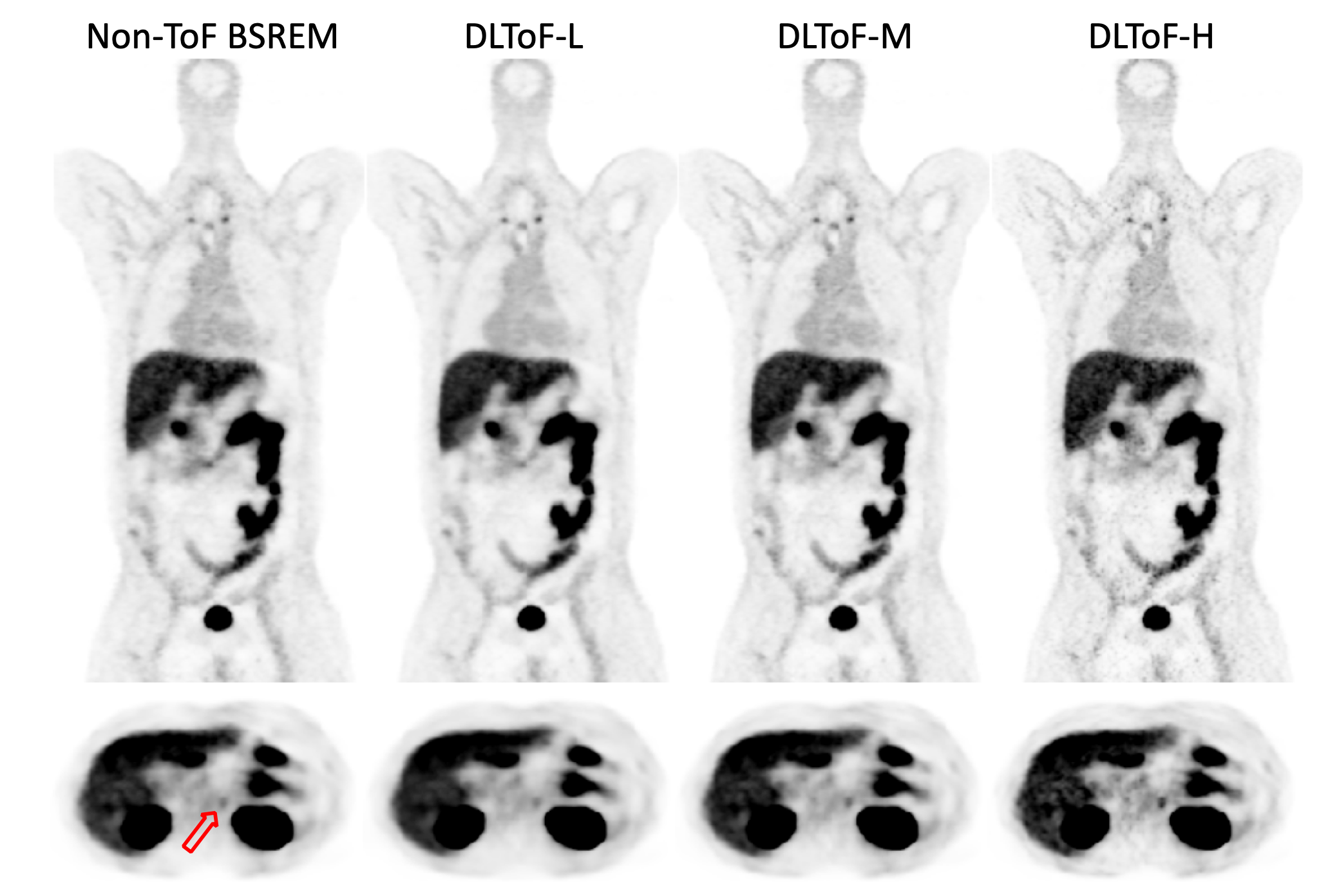


Supp Materials **Figure 4** DL-ToF enhancement of a representative ^68^Ga-PSMA test subject with a BMI of 21.8 kg/m^2^ with an injected activity of 162 MBq scanned on a GE OMNI Legend PET/CT scanner. Display window: 0-5 SUV.


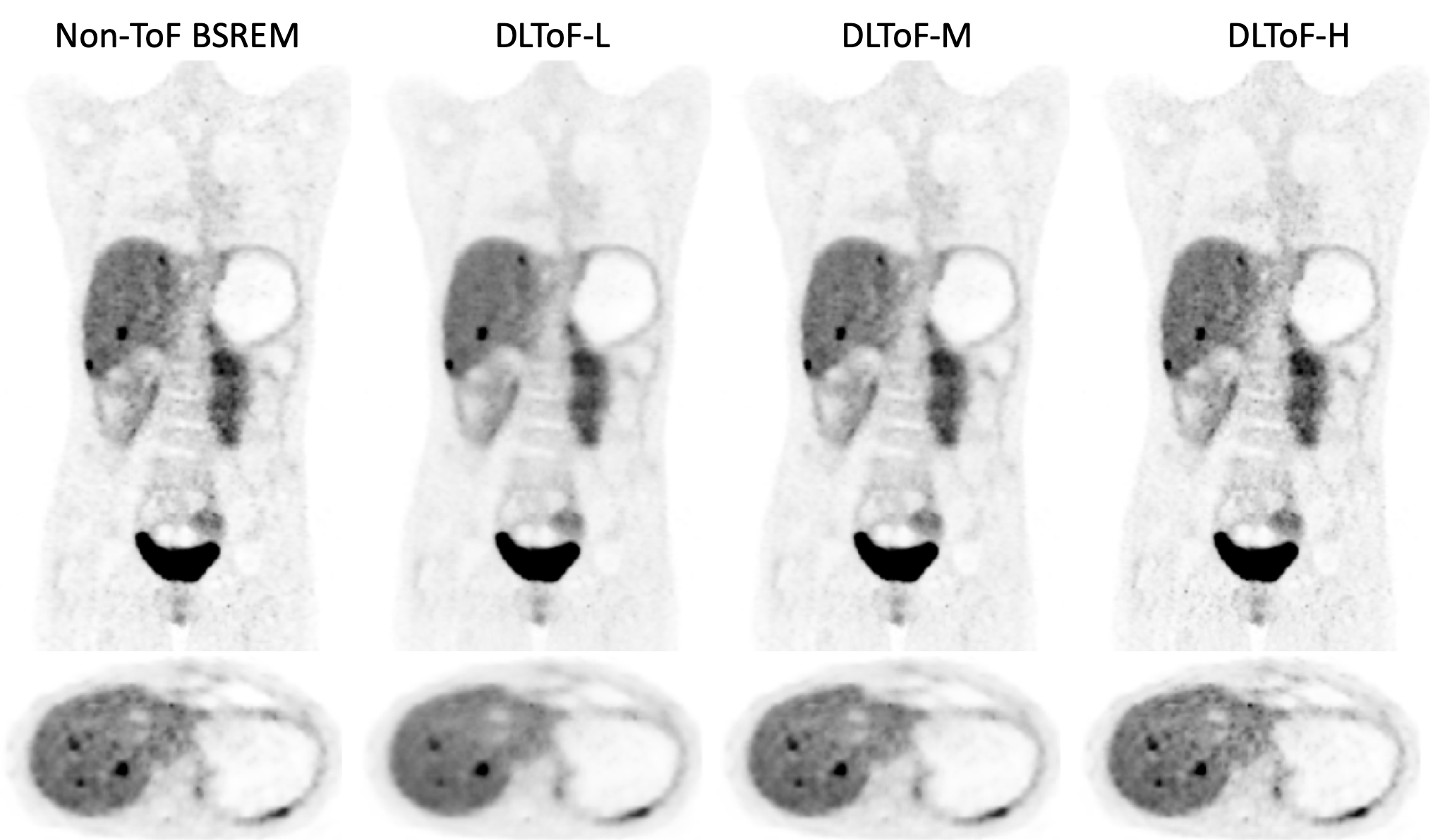


Supp Materials **Figure 5** DL-ToF enhancement of a representative ^68^Ga-DOTATATE test subject with a BMI of 18.4 kg/m^2^ with an injected activity of 192.4 MBq scanned on a GE Omni Legend PET/CT scanner. Display window: 0-5 SUV.

Supp Materials **Figure 6** Comparison of DLToF-M FDG-only single-tracer (ST) and multi-tracer (MT) versions for a representative ^18^F-FDG test subject scanned on a GE DMI PET/CT scanner. Display window: 0-5 SUV.

Supp Materials **Figure 7** Comparison of DLToF-M FDG-only single-tracer (ST) and multi-tracer (MT) versions for a representative ^18^F-PSMA test subject scanned on a GE DMI PET/CT scanner. Display window: 0-5 SUV.

Supp Materials **Figure 8** Comparison of DLToF-M FDG-only single-tracer (ST) and multi-tracer (MT) versions for a representative ^68^Ga-PSMA test subject scanned on a GE DMI PET/CT scanner. Display window: 0-5 SUV.

Supp Materials **Figure 9** Comparison of DLToF-M FDG-only single-tracer (ST) and multi-tracer (MT) versions for a representative ^68^Ga-DOTATATE test subject scanned on a GE DMI PET/CT scanner. Display window: 0-5 SUV.

Supp Materials **Figure 10** Blinded clinical reader study on a subset of 6 DMI ^18^F-FDG exams comparing DLToF FDG-only single-tracer (ST) and multi-tracer (MT) versions. The scores were done by our most experienced reader (KMB).
